# Heterogeneous Effects of Skill Training on Rural Livelihoods around Four Biosphere Reserves in China

**DOI:** 10.3390/ijerph191811524

**Published:** 2022-09-13

**Authors:** Qi Sun, Yunli Bai, Chao Fu, Xiangbo Xu, Mingxing Sun, Baodong Cheng, Linxiu Zhang

**Affiliations:** 1 School of Economics and Management, Beijing Forestry University, Beijing 100083, China; 2 Key Laboratory of Ecosystem Network Observation and Modeling, Institute of Geographic Sciences and Natural Resources Research, Chinese Academy of Sciences, Beijing 100101, China; 3 United Nations Environment Programme-International Ecosystem Management Partnership (UNEP-IEMP), Beijing 100101, China

**Keywords:** skill training, income, protected area, agricultural, off-farm

## Abstract

The growing contradiction between protection and livelihood is a common challenge for most protected areas in developing countries. Skill training is an important way to increase household income and alleviate the dilemma between conservation and development. However, its effects on household income around protected areas have rarely been explored. This paper aims to evaluate the effect of skill training on the income of households around four Biosphere Reserves in China and explore its mechanism. Based on the information collected from 381 households through face-to-face interviews, this study adopted descriptive analysis and multiple regression to yield consistent results. The results showed that agricultural and off-farm skill training had no impact on the total household income. The results from the mechanism analysis found that participation in off-farm skill training had a significant and positive effect on the total income of the households outside protected areas and participation in agricultural training had a positive effect on agricultural income. The findings indicate that the local government and protected area administration should increase the publicity for skill training, enrich the types training, appropriately supply livelihood support projects that reconcile conservation and development, and strengthen the infrastructure development around protected areas to promote off-farm employment and the circulation and sale of agricultural products. However, the impacts of any associated intensification should be carefully monitored.

## 1. Introduction

Protected areas (PAs) are important districts for global ecosystems and biodiversity conservation [1]. The International Union for Conservation of Nature (IUCN) defined a PA as an area of land and/or sea especially dedicated to the protection and maintenance of biological diversity, and of natural and associated cultural resources, and managed through legal or other effective means [2]. The global PA network covers a land area of more than 17 million km^2^, representing almost 15% of the Earth’s terrestrial area and nearly achieving the goal of the 2010 Aichi Targets of the Convention on Biological Diversity to protect 17% by 2020 [3]. PAs not only have a function of protecting wild animal and plant resources but also play a key role in improving the regional ecological environment and maintaining national ecological security. Therefore, they are important components of the global conservation strategy [4,5].

However, the global network of PAs failed to fully achieve its biodiversity conservation objectives for 2020. About 20% of threatened species were identified as “gap species” with no protection [4,6]. Furthermore, one third of the world’s PAs are heavily degraded [7]. A growing contradiction between protection and livelihood improvement has been considered as one of the main drivers for the failure [8]. Most PAs strictly constrain the use of natural resources for surrounding communities, leading to the illegal exploitation of resources by residents [9]. As PAs are mainly located in relatively poor areas, where economic development largely depends on using local natural resources, such as forest and land [9,10,11], the contradiction between protection and development is becoming increasingly serious [12,13,14].

Many authors have explored the solutions to the contradiction between conservation and development. Participatory conservation, defined as a dynamic process that responds to the changes in human needs, as well as the environment, is currently one of the most popular approaches to PA management in the world [15,16,17,18,19]. Monetary compensation policy can reduce the contradiction between PAs and communities, because it can solve the negative externality of protection contradiction and the imbalance between protection costs borne by residents and associated economic benefits [20,21]. Some PA management agencies have provided job opportunities to local residents [22,23,24,25,26] or supported the development of ecological nature-based tourism [16,27,28] to alleviate the dilemma between conservation and development.

Remarkably, few scholars have paid attention to the effect of human capital enhancement in encouraging local livelihoods to alleviate dependence on natural resources around PAs [29,30,31,32]. As an important method of improving human capital, skill training can fundamentally improve the income of local residents, as has been confirmed by numerous studies [33,34,35,36,37,38]. In the agricultural sector, for example, trained farmers make a modest gain in knowledge and professional skills, leading to reduced pesticide use [39], mitigation of human-wildlife conflicts [40], and increases in household income [41]. Thus, skill training may contribute to alleviating the contradiction between conservation and development.

The literature suggests that there is a relationship between skill training and increases in household income, but there are still obvious research gaps that need to be narrowed. First, recent studies have mainly examined the trade-offs between the effects of conservation and development interventions implemented around PAs from a macro perspective. Studies that assess the effect of interventions for human capital development—e.g., skill training—on livelihood improvement from a micro perspective are rather rare. However, the findings from micro perspectives are always problem-oriented and helpful for policy making. Second, although a number of studies have assessed the effect of skill training on household income in rural areas, few have focused on the effect in areas around PAs, the key areas for the contradiction between protection and development. Third, the mechanism underlying the effect of skill training on household income around PAs is yet to be explored, although it is important for showing the focus of the policy.

Therefore, this study examined the effect of skill training on household income and explored its mechanism in rural areas around four Biosphere Reserves in China. First, we investigated the impact of two types of skill training on household income, respectively. Second, we explored the mechanism by estimating the impacts of the two types of skill training on different types of household income within and outside PAs and, thus, analyzed the heterogeneities of the impacts. Harnessing primary information from 381 households collected through face-to-face interviews around these reserves in 2018, we used descriptive analysis and multiple regression models to carry out the research.

This study makes two potential contributions to the existing literature. First, the roles of off-farm skill training and agricultural skill training in improving livelihood around PAs are assessed from a micro perspective, which will enrich the literature associated with sustainable management of PAs. Second, the mechanism of skill training’s impact on household income, both inside and outside Pas, is explored, which will provide detailed policy implications for the alleviation of the contradiction between conservation and development by improving human capital for equal development around PAs.

The rest of the paper is structured as follows. Section 2 provides the literature review and conceptual framework of this study. Section 3 introduces the methodology, including sampling and data collection, variables, and model specification. Section 4 presents the results of descriptive analysis. Section 5 describes the empirical results from the multiple regression. Section 6 provides the conclusion and policy implications.

## 2. Literature Review and Conceptual Framework

Households require a range of assets (including human capital, natural capital, financial capital, physical capital, and social capital) to achieve positive livelihood outcomes. They have to seek ways of nurturing and combining the assets they do have to ensure survival [42,43]. The human capital of a household usually comprises the number of laborers and their educational attainment, skill acquisition, and the health conditions of household members. The health conditions are highly correlated with the ages of the family members [30,44]. Natural capital is mainly indicated by the area of land owned or operated by a household; specifically, arable land and forest land [45,46]. Financial capital is measured by the accessibility of inclusive finance [8,30]. Physical capital is indicated by the accessibility of infrastructure and equipment, such as roads [29]. Social capital is usually measured by membership in parties or organizations [30,46].

The accessibility to the above types of capital determines the choice of a household’s livelihood strategies [46]. Specifically, the households around PAs generally use these types of capital to obtain income through the strategies such as farming and non-farm activities [47]. Many scholars have demonstrated the decisive role of human capital on household income level and growth [48,49,50]. As an important form of livelihood capital facilitating the improvement of the utilization efficiency of other types of capital, human capital has played an important role in increasing income for residents around PAs by improving the overall efficiencies of livelihood strategies [44,46,51]. Investment in human capital might help to diversify livelihood means and, thus, dependency on nature resources would be reduced [30].

Skill training, as an important means of improving human capital, has a significant impact on off-farm income growth. Attanasio et al. [37] evaluated the long-term impacts of a Colombian randomized training and job placement program and found that it had continuous effects increasing the probability of formal employment, earnings, and the probability of working in large firms. Lee [34] assessed the wage effects of the Job Corps program, one of the largest federally funded job training programs in the U.S., and suggested that the program raised earnings of participants in the program by increasing their human capital. In China, skill training had positive effects on the participation rate in off-farm employment for rural laborers and their wages [33]. The positive effect of skill training on the performance of the labor market was also found in New Zealand [52] and the UK [53].

The effect of skill training in the agricultural sector has been explored widely. For instance, Schreinemachers et al. [41] used farm-level data from 94 trained and 151 non-trained farm households and found that, for the average smallholder vegetable farmer, planting skill training increased net household income by about 48%. Chesterman et al. [54] estimated the socio-ecological effects of a soil and water conservation training program for farmers implemented by an Ethiopian non-governmental organization and found that participants in training sessions had a higher income from agriculture than non-participants. Furthermore, in the hillside areas in Honduras, there was a large and statistically significant positive association between general agricultural training and household income [29]. However, there is little research on the role of skill training on household income in the areas around PAs, where the effectiveness of training has yet to be verified.

**Figure 1:**
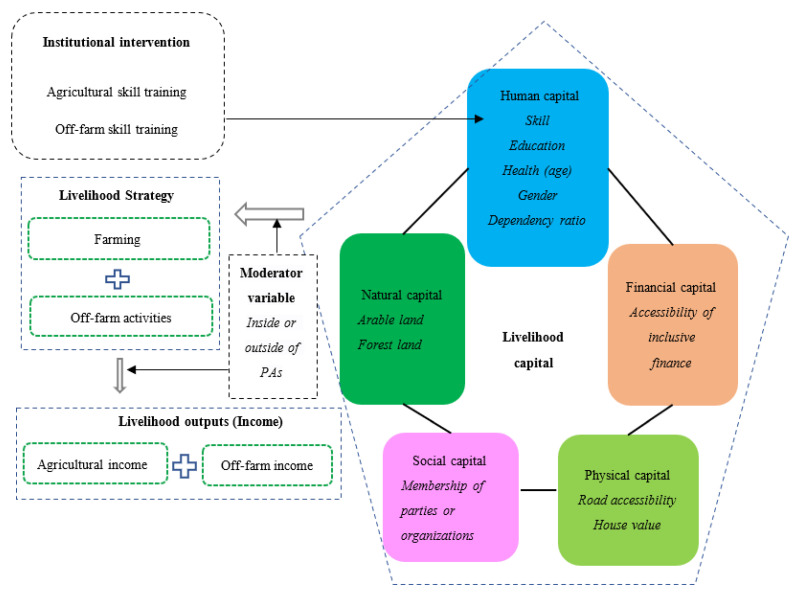
Conceptual framework of this study.

Based on the above analysis, the following conceptual framework was developed for this study (Figure 1). The local government aims to reduce the pressure on natural resources around PAs by improving the income of surrounding households through agricultural skill training and off-farm skill training (institutional intervention). Such training affects the human capital of these households through certain other types of livelihood capital, which can influence livelihood strategies and further lead to a change in household income. However, the impact of skill training on income may differ between the households inside and outside of the PAs. considering the management features within the boundary of the areas selected for conservation. These logical analyses are shown in the conceptual framework of this study (Figure 1). Under the guidance of this framework, the empirical model was established, as described in the next section.

## 3. Materials and Method

### 3.1. Study Sites and Sampling

**Figure 2:**
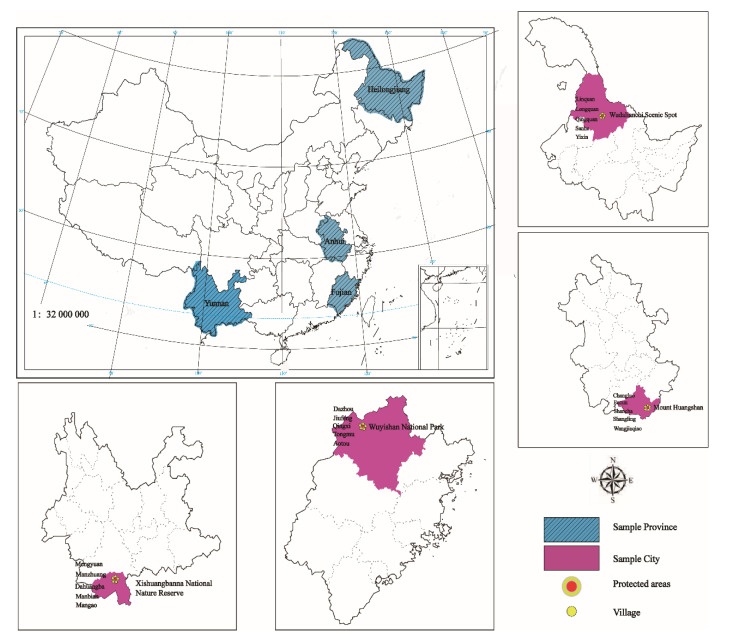
Study sites around four Biosphere Reserves in China.

The study used the primary data collected by the authors in April 2018 at the sites around four Biosphere Reserves in China, including the Xishuangbanna National Nature Reserve, Mount Huangshan Scenic Area, Wuyishan National Park, and Wudalianchi Scenic Area and Nature Reserve (Figure 2). Biosphere Reserves are multipurpose PAs designated by the intergovernmental Man and the Biosphere (MAB) Program to promote sustainable development [55]. They promote solutions reconciling the conservation of biodiversity with its sustainable use in diverse ecological, social and economic, and institutional contexts. The four Biosphere Reserves selected in this study span distinct climatic types, economic development levels, and PA management systems in China.

The Xishuangbanna National Nature Reserve is located in Xishuangbanna Dai Autonomous Prefecture in the south of Yunnan Province. Accompanied by a tropical humid climate, it is a large-scale, comprehensive nature reserve, with the main purpose of protecting tropical the forest ecosystem and rare wild animals and plants [56]. It is the largest PA in China, with the most comprehensive tropical forest and extremely rich biological resources. There are a large number of rare wild animals and plants, including rare and endangered species such as Asian elephants. In 1993, it was accepted by the MAB Program as a member of the World Network of Biosphere Reserves. For three decades, the community condominium, a community-based approach to conservation, has been implemented by the Reserve’s Management and Conservation Bureau (MCB), affiliated to the Forestry and Grassland Bureau of Xishuangbanna Prefecture, and surrounding villages to balance ecosystem conservation and livelihood improvement. Each village around the Reserve has established a leading group, consisting of the MCB staff, the town mayor, village cadres, forest rangers, and women from the village. As the result of constant consultation and negotiation between the MCB and villagers, an agreement on the community condominium was developed to stipulate the rights and obligations of villagers in using natural resources. The MCB and local government have also implemented a variety of skill training programs in the villages, under themes such as understory planting for traditional Chinese medicine, tea processing, and bee-keeping, to improve the livelihood of villagers.

The Mount Huangshan Scenic Area is located in South Anhui Province and accompanied by a subtropical monsoon climate and an evergreen broad-leaved forest, with a forest coverage rate of more than 80% [57]. Mount Huangshan is a scenic area rather than a nature reserve, giving it more rights to develop tourism. With the rapid development of tourism, the surrounding villagers were encouraged to run businesses or become wage earners around the area. In recent years, the Huangshan Scenic Area Management Committee (HMC), affiliated with the Huangshan Municipal People’s Government, has become aware of the importance of protecting the environment for its sustainable development. With intensified efforts to protect ecosystems, Mount Huangshan Scenic Area successfully joined the MAB Program in 2018. It can be seen that Mount Huangshan Scenic Area underwent a process of development before protection, which is quite different from that of nature reserves. To balance ecosystem conservation and livelihood improvement for villagers, the HMC built a kiwi planting base and a cattle breeding center to train villagers, provided ecological rewards to them, and invested in building roads and fireproofing facilities in villages. Due to the cumulating development of rural tourism and community services, the local residents have gradually transformed from traditional agricultural practices, which involve heavy physical labor and resource consumption, to rural tourism service and ecological and leisure agriculture.

The Wuyishan National Park, spanning Jiangxi and Fujian provinces, joined the MAB Program in 1987 when it was a national nature reserve. Located in the inland mountainous area, the Park has a mid-subtropical monsoon climate, an evergreen broad-leaved forest, a coniferous and broad-leaved mixed forest, and other vegetation types [58]. Unlike Xishuangbanna and Mount Huangshan, the Wuyishan National Park Administration (WNPA, the former Wuyishan National Nature Reserve Administration), affiliated with the Government of Fujian Province, has adopted the strategy of “10% production promoting 90% protection” since the 1980s. Under this strategy, about 10% of the reserve’s experimental area was designated to produce bamboo and tea to solve the livelihood problems of villagers. Additionally, small-scale under-forest breeding can be carried out in the experimental area. The subsidy for the public welfare forest in the reserve is 3 yuan per mu, more than that outside the reserve. A coordination department was developed under the WNPA to be responsible for coordinating community development and ecological protection, mainly through implementing projects on garbage disposal subsidies, the under-forest economy, and ecological tea gardens, all of which were directly associated with livelihood improvements for local communities.

The Wudalianchi Scenic Area and Nature Reserve is located in Heilongjiang Province and is affiliated with the Heihe Municipal People’s Government. It joined the MAB Program as a Biosphere Reserve in 2003. Wudalianchi volcano, the main body of the reserve, is one of the most famous young volcanic groups in China, with a period of only 200 years since its last eruption. Accompanied by a temperate continental monsoon climate, the reserve is composed of mountains, water body, rocks, and springs, and it is known as the most recent volcanic area, with the richest volcanic landscape and the most detailed historical records, in China [59]. In order to protect ecosystems in the area, the Wudalianchi Scenic Area and Nature Reserve Management Committee (WMC) have implemented the “Grain for Green” Program since 2006. Ecological migration and financial investment were carried out to protect the lake and grassland in the reserve. Taking advantage of its designation as a scenic area, the development of tourism has brought many off-farm jobs for local residents. To transform the livelihoods of ecological migrants, the WMC regularly organizes free skill training programs and encourages and supports participants to engage in self-employed businesses, such as tour guides, taxis operations, home hotels, and catering services.

At each site, we selected five villages randomly. They were selected from the candidate villages located within or adjacent to the reserve boundary. These candidate villages were evenly divided into five groups according to per capita income in 2017, and then one village was selected at random from each group. A total of 20 villages were finally selected for the study, including nine located within the reserves, nine outside, and two crossing the reserve boundaries.

Rural households were randomly selected from each village. For each selected village, the village cadres were first interviewed to get the household register list, from which 20 households were randomly selected for the survey. As a result, 400 rural households were surveyed by face-to-face interview. Due to missing data, the information for 381 households was finally used as the sample of this study. Each of these four reserves can be generally divided into three parts: a core area, a buffer zone, and a transition area. According to the national and local regulations on PAs, human activities, including scientific research, monitoring, training, and education, are only allowed in the buffer zone or transition area. Therefore, the study defined the households within the buffer zone or transition area as “within PA” and those outside the reserves as “outside PA”. In the Mount Huangshan Scenic Area, however, there were no households within the whole area when this study surveyed it (just before it joined the MAB Program and expanded to include buffer zones in the reserve). Therefore, we defined the households located within 7 km of the boundary of Mount Huangshan Scenic Area (the average distance from households to the boundary in the samples) as “within PA” and those further than 7 km as “outside PA”. Among the 381 households, 158 were located “within PA”, and 223 of them were “outside PA”. The distribution of samples is shown in Table 1.

### 3.2. Data Collection

Rich information was collected at three levels; i.e., from the administration offices of each Biosphere Reserve, village, and household. The information provided by the administration offices included the reserve’s basic conditions, its organizational structure and management system, protection measures and inputs, community development measures and investments, conflict resolution mechanisms, and so on. Information provided by the village cadres included the village’s demographic structure and infrastructure, transfer payments received (including ecological compensations), community development measures and funding sources, and the relationship with the reserve administration office. The information at the administration-office and village levels was mainly gathered through focus group discussion and key informant interviews.

The authors designed the household questionnaire and trained the interviewers in accordance with the conceptual framework of this study. The information collected at the household level included the individual characteristics of each family member, as well as the human capital, financial capital, physical capital, social capital, natural capital, livelihood activities, household income, and skill training for each family member who participated. The detailed information collected from the sampled households is shown in Table 2.

### 3.3. Model Specification

For this study, a reduced form of income function was built using ordinary least squares (OLS) models to examine the effect of skill training on rural household income around these four Biosphere Reserves. The model was specified as follows:

(1) $$\ln Y_{i}=\alpha +\beta T_{i}+\gamma R_{i}+\delta H_{i}+\eta S_{i}+\zeta P_{i}+\theta N_{i}+\varepsilon_{i}$$

In Equation (1), $Y_{i}$ indicates the income of the household i, including the per capita total income, per capita off-farm income, and per capita agricultural income of the household. T is a vector of two dummy variables, including Agritech and Offfarmtech, which indicates the type of training the household participated in. Specifically, Agritech = 0 means the household did not participate in agricultural skill training, and Agritech = 1 means the household participated in agricultural skill training. Offfarmtech = 0 means the household did not participate in off-farm skill training, and Offfarmtech = 1 means the household participated in off-farm skill training. Similarly, R is a vector of three dummy variables indicating whether the household is located in the PA. Mount Huangshan = 0 means the household is not located in this PA, Mount Huangshan = 1 means the household is located in this PA. Wuyishan National Park and Wudalianchi Scenic Area Nature Reserve are the same. H represents the vector of the human capital variables, including the household head’s age, gender, and education level and the household dependency ratio. S represents the vector of the social capital variables, including whether there are Chinese Communist Party members in the household and whether there are village cadres in the household. P represents the vector of the physical capital variables, including house value and road accessibility; that is, whether there is an asphalt/cement road passing through the village. N represents the vector of the natural capital variables, including forest land area and farmland area. β represents the semi-elastic coefficients that capture the effects of different types of skill training on household income. γ, δ, η, ζ, and θ are the vectors of the semi-elastic coefficients measuring the effects of other control variables on household income, respectively. α is the constant term, and $\varepsilon_{i}$ is the error term. The description of explanatory variables is shown in Table 2.

In the model, there are three dependent variables, which are the log of the per capita total household income, the log of the per capita off-farm income of the household, and the log of the per capita agricultural income of the household. The key independent variables are the proportion of households participating in agricultural skill training and in off-farm skill training, with mean values of 13% and 7%. There are also a series of control variables in the model. For human capital, the average age of the household head was 54, and 92% of households were male-headed. Only 11% of household heads had received a junior high school or higher education. The mean of the dependency ratio was 31.59 among these households. As for social capital, 22% of households included Chinese Communist Party members and 10% of households included village cadres. For physical capital, the mean of the log of the house value was 2.7. Ninety percent of households located in the villages had asphalt/cement roads passing through them. For natural capital, the average area of forest land and farmland associated with these households was 24.75 mu and 13.25 mu, respectively. As for the reserve categorical variable, the sample proportions for Mount Huangshan, Wuyishan National Park, and Wudalianchi Scenic Spot and Nature Reserve were 25%, 25%, and 24%, respectively.

## 4. Results

### 4.1. Descriptive Results

**Figure 3:**
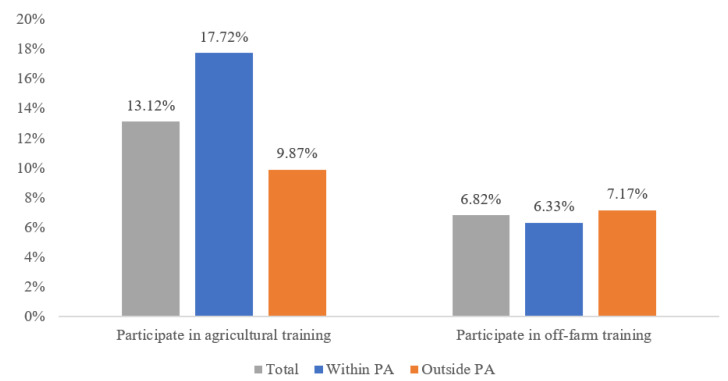
Proportion of households participating in skill training (%).

The proportion of households around the four Biosphere Reserves participating in agricultural skill training was significantly higher than the proportion participating in off-farm skill training. Specifically, 13.12% and 6.82% of households participated in agricultural and off-farm skill training, respectively. Further, the proportions of participation varied within and outside the PAs. It is notable that 17.72% of households within the PAs attended agricultural skill training in contrast to only 9.87% outside the PAs. Households were more inclined to engage in agriculture-related activities due to the abundance of natural capital within the PAs [60]. However, there was no significant difference between the proportions of participation in off-farm training within and outside the PAs, which were 6.33% and 7.11%, respectively (Figure 3).

**Figure 4:**
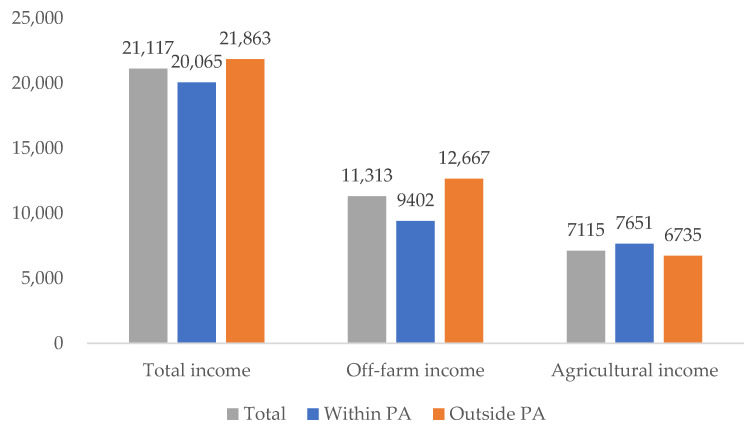
Different income types for households at study sites (CNY).

There was no significant difference in per capita income between the households within and outside PAs, but the compositions of their income differed (Figure 4). The average per capita income of households around the PAs was 21,117 CNY in 2018, which was higher than the national level of 14,617 CNY in the same year [61]. The average per capita income of the households within PAs was 20,065 CNY, slightly lower than that outside PA. However, the gap was not significant even at a 10% confidence level. Chinese household income usually encompasses five sources, including wage income, operating income, property income, transfer income, and other income. Operating income includes agricultural income and off-farm income. The main source of wage income comes from the non-agricultural sector. Transfer income mainly refers to government subsidies and the value of social or private donations received by families [62,63]. With the expansion of off-farm employment in rural households, off-farm earnings became the primary source of household income around PAs, which is similar to other areas in China [61]. Although off-farm earnings were the leading source of household income, their proportions in the household income differed between the households within and outside PAs. The proportion of off-farm income in the household income was 24.4% for the households within PAs which was 14 percentage points less than those outside PAs.

**Figure 5:**
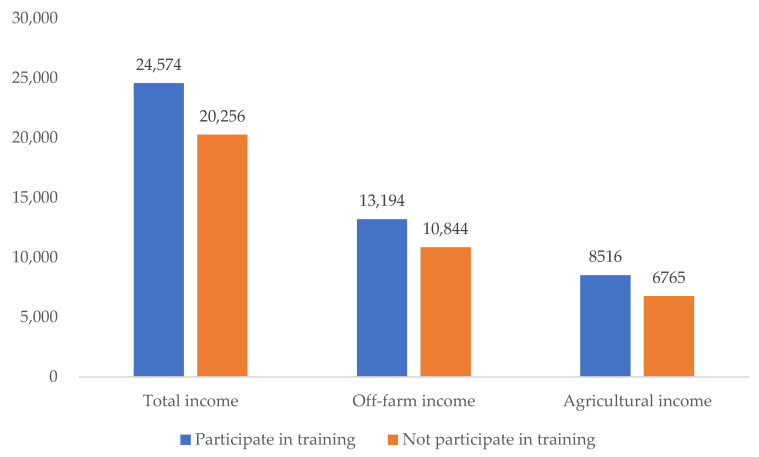
Income of households participating in skill training and not participating in skill training (CNY).

Households who participated in skill training had obviously higher income than those who did not participate in any training (Figure 5). It can be seen that the per capita agricultural income and per capita off-farm income of the participants were 20% higher than those of non-participants. This finding indicates that skill training, as an important means to develop human capital, can play a crucial role in increasing the income of households around PAs, similarly to other regions in China [64].

**Figure 6:**
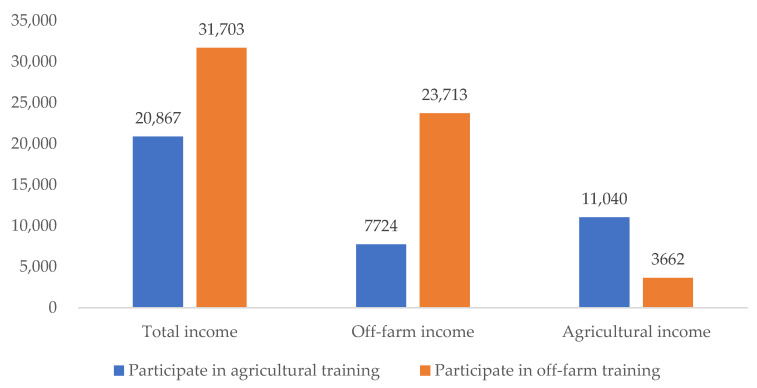
Income of households participating in different types of training (CNY).

The income of these households seemed to be related to the types of skill training they participated in. The average per capita income of the households participating in off-farm skill training was 31,703 CNY, which was nearly 1.5 times that of those participating in agricultural skill training. As for off-farm income, the households participating in off-farm skill training had a per capita income of 23,713 CNY, while for households participating in agricultural skill training it was 7724 CNY. As for agricultural income, the households participating in agricultural training had a per capita income up to 11,040 CNY, which was significantly higher than those participating in off-farm skill training (Figure 6). The aforementioned differences in the two kinds of income were both significant at a 1% confidential level.

### 4.2. Empirical Results

The empirical results are presented in Table 3, Table 4, Table 5 and Table 6. The models performed well and the results were generally consistent. The effects of most control variables were also as expected. The robustness of the models was tested by gradually adding control variables. For brevity, we only report the results yielded from the most robust models, which include most control variables.

#### 4.2.1. The Impact of Skill Training on Per Capita Income

According to the regression results, neither agricultural nor off-farm skill training had a significant impact on the total household income (Table 3). Did the training work to improve household income within and outside the PAs? When looking at the effects of skill training on the income of the households within and outside the PAs, respectively, it can be seen that only participating in off-farm skill training had a significant and positive effect on the per capita income of the households outside the PAs. When households outside the PAs participated in off-farm skill training, their per capita income was 45.4% higher than that of the households not participating in any training (*p* < 0.05) (Table 4).

Other factors affected the household income around PAs in this study; for example, the higher the dependency ratio, the lower the income (*p* < 0.01). A high dependency ratio means few laborers and many children and elders in households, which results in low per capita income. Physical capital had a significant and positive effect on the income of households both within and outside PAs. Specifically, if the house value increased by 1%, per capita income rose by 9% and 17.4% for households within and outside PAs, respectively (*p* < 0.01). Whether there is an asphalt or cement road passing through a village had no significant impact on the income of households outside PAs, but it increased the income of households within PAs by 90.8% (*p* < 0.01). This reaffirms that road accessibility increased the opportunities for residents inside PAs to obtain higher incomes [25]. The per capita income of households outside PAs increased by 0.5% if their forest land area increased by 1 mu (*p* < 0.01). Similarly, the per capita income of households within and outside PAs increased by 0.8% and 0.6%, respectively, if their farmland area increased by 1 mu (*p* < 0.01). Among the four Biosphere Reserves, only households outside Mount Huangshan had significant differences in per capita income compared to Xishuangbanna National Nature Reserve, on average 34.5% higher than the latter (*p* < 0.05).

#### 4.2.2. The Mechanism of the Impact of Skill Training on Income

##### The Impact of Skill Training on Agricultural and Off-Farm Income

According to the results of the OLS estimation, neither agricultural nor off-farm skill training had a significant impact on the per capita off-farm income of households around PAs (Table 5). However, participation in agricultural skill training, on average, had a positive effect on the per capita agricultural income (*p* < 0.01). The per capita agricultural income of the households participating in agricultural skill training was 69.5% higher than that of the households with no training.

Some categories of human capital were correlated with the off-farm income and agricultural income of the households around PAs. Age was negatively related with off-farm income (*p* < 0.05), but it had no effect on agricultural income. The elder rural laborers were less likely to be employed or earned less in the off-farm labor market [65], but they showed no significant difference in their agricultural income due to the universal aging of agriculture in China [66]. The households with male heads were more likely to have high agricultural incomes (*p* < 0.1). The household dependency ratio mainly affected off-farm income, rather than agricultural income, in the households around PAs. The development of agricultural technology leads to labor surpluses in the agricultural sector [67], which may have resulted in the absence of a relationship between the household dependency ratio and agricultural income.

Physical capital had a significant effect on the off-farm income and agricultural income of the households around PAs. The house value had positive and negative effects on off-farm income and agricultural income, respectively. The households with high-value houses were more likely to invest in family businesses, one important type of off-farm activity, which made them more likely to earn off-farm income. Road accessibility had a positive effect on agricultural income (*p* < 0.1). Road accessibility improves market accessibility and decreases transportation costs in agriculture production [68]. As for natural capital, the size of forest land had a positive effect on agricultural income (*p* < 0.05). The size of farmland had positive effects on both off-farm income and agricultural income (*p* < 0.01).

From the reserve perspective, the households around Mount Huangshan and Wuyishan National Park had higher per capita off-farm incomes than those in Xishuangbanna National Nature Reserve (*p* < 0.01). The agricultural income of the households around the other three Biosphere Reserves was significantly lower than that around Xishuangbanna, which was closely related to the fact that local farmers in Xishuangbanna rely heavily on rubber cutting as the main source of their household income.

##### The Heterogeneity of the Impact of Skill Training on Household Income within and outside PAs

According to the above analysis, agricultural skill training had no effect on the total income of the households around PAs, but it had a positive effect on their agricultural income. Further, off-farm skill training affected the total income of the households outside PAs; however, it had no effect on their off-farm income or agricultural income. Did agricultural skill training have the same effects on the agricultural income of the households within and outside PAs? How did off-farm training affect total income other than off-farm or agricultural income for households outside PAs? To answer such questions, Table 6 presents the heterogeneity of the impact of off-farm and agricultural skill training on off-farm income and agricultural income among the households within and outside PAs, respectively.

The results of the OLS estimation show that agricultural skill training had significant and positive impacts on agricultural income both within and outside PAs (Table 6). Participation in agricultural training could increase per capita agricultural income by 77% within PAs (*p* < 0.1) and by even more than 100% outside PAs (*p* < 0.01). This indicates that the agricultural skill training had a much greater effect on agricultural income outside the PAs.

The effect of off-farm skill training only boosted the off-farm income of the households outside PAs. Among the households outside PAs, the per capita off-farm income of those participating in off-farm training was 78.3% higher than those without training (*p* < 0.1). The off-farm activities within PAs are restricted according to natural conservation and management regulations, which leads to limited off-farm employment opportunities. Although the households participated in off-farm training, they had little chance to obtain off-farm earnings.

Human capital had a great effect on the off-farm income of the households within the PAs. The off-farm income of these households decreased by 15.5% with each one-year increase in the age of the household head (*p* < 0.01). The off-farm income of the households within PAs decreased by 5.4% with each one-unit increase in the dependency ratio (*p* < 0.01). The agricultural income of the households with male heads was greater than those with female heads within PAs (*p* < 0.1), but showed no significant difference outside PAs, probably due to the fact that agricultural activities require more physical labor.

Different types of natural capital had similar effects on agricultural income in the households within and outside PAs. The per capita agricultural income increased by more than 3% when the size of farmland increased by 1 mu for both households within and outside PAs (*p* < 0.01). However, the effects of physical capital on off-farm income and agricultural income differed. It can be seen that house value had a positive effect on the off-farm income of households outside PAs but a negative impact on the agricultural income of households within PAs (*p* < 0.1). In addition, road accessibility generally had positive effects on the off-farm income among households outside PAs and the agricultural income among households within PAs (*p* < 0.01).

The households within and outside the three of the Biosphere Reserves, except for those outside Wudalianchi, had much higher off-farm incomes than their counterparts around Xishuangbanna. In contrast, they all had much lower agricultural incomes than their counterparts within and outside Xishuangbanna.

## 5. Discussion

The descriptive analysis showed that the proportions of off-farm earnings in the household income were quiet different between the households within and outside the PAs, and this was mainly determined by the dependence on natural resources and off-farm job opportunities [69,70,71]. The households around PAs strongly depended on agricultural production [72,73]. Agricultural income accounted for more than 40% of total household income among these households, which was much larger than that of the households outside the PAs [74].

The results from regressions showed that off-farm skill training had a significant and positive effect on the per capita income of the households outside PAs. This finding indicates that the households outside PAs were more likely to gain a higher per capita income by participating in off-farm skill training. Off-farm skill training plays an important role in improving farmers’ income [75], especially for households outside PAs. It had no effect on the income of households within PAs. This is probably because of relatively limited employment opportunities due to considerable restrictions on resource utilization and requirements for environmental protection within PAs [69]. Although the households participated in off-farm training, they had little chance to obtain off-farm earnings, such as earning wages in off-farm sectors or running businesses. This further demonstrates the challenge in addressing income inequality among households within and outside PAs [63].

Agricultural skill training increased agricultural income, which is consistent with previous studies [41,76]. Although participation in agricultural skill training increased agricultural income, it showed no effect on the total household income. This may have been due to its low proportion in terms of the total income.

When considering the heterogeneity within and outside PAs, it can be seen that agricultural skill training had a much greater effect on agricultural income outside PAs, and off-farm skill training only significantly increased off-farm income among households outside PAs. As for agricultural skill training, however, it had a much greater effect on agricultural income outside PAs. There are two possible reasons to explain this finding. First, the households within PAs mainly depended on agricultural income, and they had already made private investments in agricultural equipment and spent much time on self-training in agricultural skills. Thus, the agricultural skill training provided by the local governments and PA administrations had little impact on them. Second, the households outside PAs had better market accessibility than those within PAs, which made it much easier for them to sell agricultural products after receiving agricultural skill training. In addition, the restrictions on various resource utilization and livelihood activities may have resulted in limited off-farm employment opportunities within PAs [77]; as a result, off-farm training only significantly increased off-farm income among households outside PAs.

Agricultural skill training had significant effects on agricultural income both within and outside PAs. Carrying out agricultural skills training is conducive to improving farmers’ planting techniques and increasing the income level, which indirectly reduces the excessive use of natural resources and, thus, ensures the ecological balance. On the other hand, off-farm training only increased off-farm income among households outside the PAs. Strengthening off-farm training for households outside PAs could incentivize them to increase off-farm economic activities, thus further reducing the possibility of damaging the ecological system.

## 6. Conclusions

In this study, the impacts of two different types of skill training on household income were estimated using the data collected from 381 households around four Biosphere Reserves in China. An OLS model was used to obtain robust results. It was found that neither agricultural skill training nor off-farm skill training had significant impacts on total household income. However, off-farm skill training had a significant and positive effect on per capita income among the households outside PAs. Due to the greater number of restrictions on resource utilization and environmental protection requirements within PAs, there are relatively fewer employment opportunities for local residents, which may have led to the above results.

In particular, household income was classified into agricultural income and off-farm income. It can be seen that neither agricultural nor off-farm skill training had significant impacts on off-farm income. Agricultural training had a positive effect on agricultural income but no effect on the total household income, which may have been due to the low proportion of agricultural income in the total household income. Further, the study analyzed the differences in the impact of training on household income between households within and outside PAs. It was found that agricultural skill training had significant and positive impacts on agricultural income both among households within and outside PAs, with a particularly greater impact on the latter.

The findings of this study have profound policy implications. Skill training is an important factor helping farmers around PAs increase household income. It is necessary to increase the publicity for skill training, enrich the types of skill training available, and encourage more households to participate. In addition, reasonable utilization of biodiversity and other natural resources on the basis of conservation is an effective path for sustainable development. Specifically, the government and PA administrations should appropriately supply livelihood support projects reconciling the conservation and sustainable use of natural resources, such as ecological agriculture and tourism, to increase off-farm employment opportunities for local residents within PAs. Moreover, local governments and reserve administrations should further strengthen infrastructure development, especially the improvement of roads around PAs, to promote off-farm employment and the circulation and sale of agricultural products. However, the impacts of any associated intensification should be carefully monitored, considering the possible unintended negative consequences of accentuating anthropogenic pressures on PA ecosystems.

There are still some shortcomings in this study. First, financial capital was not included in the control variables due to its low level and lack of variation in the samples. Second, the effect of skill training under the different management systems of the four Biosphere Reserves is still unclear and should be further explored in the future to provide more specific policy suggestions for PA administrations. Third, this study only collected data from one period. If there is funding support in the future, further research will be conducted, such as focusing on the effects of skill training after the COVID-19 pandemic and the influencing factors for farmers’ livelihood strategies around PAs and the impact on poverty.

## Figures and Tables

**Table 1:** Sample distribution in this study.

Biosphere Reserves	Number of Villages	Number of Households	Number of Households within PA	Number of Households outside PA
Xishuangbanna National Nature Reserve	5	99	40	59
Wuyishan National Park	5	95	43	52
Mount Huangshan Scenic Area	5	95	20	75
Wudalianchi Scenic Area and Nature Reserve	5	92	55	37
Total	20	381	158	223

**Table 2:** Descriptive statistics of the samples.

Variable	Definition	Mean	Std. Dev.	Min	Max
Training					
Agritech	Participation in agricultural skill training	0.13	0.34	0	1
Offfarmtech	Participation in off-farm skill training	0.07	0.25	0	1
Income					
Totalincome	Household income (CNY)	85,341.97	84,997.14	2921	673,000
Perincome	Per capita household income (CNY)	21,117.42	21,566.08	1033.33	240,000
Peroffincome	Per capita off-farm income of the household (CNY)	11,313.05	16,970.54	0.1	150,000.1
Perfarmincome	Per capita agricultural income of the household (CNY)	7114.54	12,178.73	0.01	138,600
lnperincome	Log of per capita household income	9.61	0.85	6.94	12.39
lnperoffincome	Log of per capita off-farm income of the household	5.87	5.24	−2.30	11.92
lnperfarmincome	Log of per capita agricultural income of the household	7.26	3.04	−5.30	11.84
Human capital					
Age	Age of the household head	54.35	11.79	23	84
Gender	Gender of the household head (1 = male; 0 = female)	0.92	0.28	0	1
Education	Whether the education level of the household head is above junior middle school (1 = yes; 0 = no)	0.11	0.31	0	1
Perfeed1	Household dependency ratio (%)	31.59	30.31	0	100
Social capital					
Party	Whether there are Chinese Communist Party members in the household (1 = yes; 0 = no)	0.22	0.42	0	1
Cadre	Whether there are village cadres in the household (1 = yes; 0 = no)	0.10	0.30	0	1
Physical capital					
lnHouseValue	Log of house value	2.70	1.54	−4.61	6.21
Road	Whether there is an asphalt/cement road passing through the village (1 = yes; 0 = no)	0.90	0.30	0	1
Natural capital					
Forestland	Forest land area (mu)	24.75	35.65	0	300
Farmland	Farmland area (mu)	13.25	24.15	0	213.6
Biosphere Reserves ^a^					
Mount Huangshan	Whether the household is located in this PA (1 = yes; 0 = no)	0.25	0.43	0	1
Wuyishan National Park	Whether the household is located in this PA (1 = yes; 0 = no)	0.25	0.43	0	1
Wudalianchi Scenic Spot and Nature Reserve	Whether the household is located in this PA (1 = yes; 0 = no)	0.24	0.43	0	1

Note: ^a^ Xishuangbanna National Nature Reserve is the reference group.

**Table 3:** The impact of skill training on total household income.

Variables	Dependent Variable: Log of per Capital Household Income		
	Model 1	Model 2	Model 3
Training			
Agritech	−0.022	−0.115	−0.100
	(−0.149)	(−1.016)	(−0.854)
Offfarmtech	0.329 *	0.274 *	0.197
	(1.698)	(1.937)	(1.520)
Human capital			
Age		0.006 *	0.001
		(1.920)	(0.410)
Gender		0.023	−0.036
		(0.146)	(−0.227)
Education		−0.100	−0.145
		(−0.914)	(−1.250)
Perfeed1		−0.007 ***	−0.007 ***
		(−5.556)	(−5.493)
Social capital			
Party		0.186 *	0.148
		(1.923)	(1.544)
Cadre		0.201	0.221 *
		(1.642)	(1.864)
Physical capital			
lnHouseValue		0.176 ***	0.155 ***
		(6.281)	(5.225)
Road		0.353 ***	0.144
		(2.625)	(1.012)
Natural capital			
Forestland		0.005 ***	0.006 ***
		(5.595)	(5.053)
Farmland		0.006 ***	0.007 ***
		(6.732)	(6.071)
Biosphere Reserves			
Huangshan			0.485 ***
			(4.269)
Wuyishan			0.302 ***
			(2.619)
Wudalianchi			0.216 *
		8.413 ***	(1.668)
Constant	9.588 ***	(35.600)	8.719 ***
	(204.732)		(34.959)
Observations	381	381	381
R squared	0.01	0.356	0.383

Note: * and *** denote significant mean differences at the 10 and 1 percent levels.

**Table 4:** The impact of skill training on per capita household income within and outside PAs.

Variables	Dependent Variable: Log of per Capita Household Income	
	Within PA	Outside PA
Training		
Agritech	−0.021	−0.101
	(−0.124)	(−0.575)
Offfarmtech	−0.078	0.454 **
	(−0.554)	(2.376)
Human capital		
Age	−0.006	0.007
	(−1.056)	(1.636)
Gender	−0.24	0.088
	(−1.125)	(0.392)
Education	−0.033	−0.084
	(−0.146)	(−0.615)
Perfeed1	−0.006 ***	−0.006 ***
	(−2.970)	(−3.687)
Social capital		
Party	0.092	0.172
	(0.698)	(1.315)
Cadre	0.141	0.244
	(0.815)	(1.566)
Physical capital		
lnHouseValue	0.090 **	0.174 ***
	(2.064)	(4.150)
Road	0.908 ***	−0.311
	(3.670)	(−1.605)
Natural capital		
Forestland	0.000	0.005 ***
	(0.240)	(3.430)
Farmland	0.008 ***	0.006 ***
	(4.119)	(3.789)
Biosphere Reserves		
Huangshan	0.580 **	0.345 **
	(2.540)	(2.493)
Wuyishan	0.550 ***	0.108
	(3.141)	(0.662)
Wudalianchi	−0.134	0.209
	(−0.580)	(1.178)
Constant	8.955 ***	8.738 ***
	(20.054)	(31.339)
Observations	158	223
R squared	0.559	0.344

Note: ** and *** denote significant mean differences at the 5 and 1 percent levels.

**Table 5:** The impacts of two different types of skill training on household off-farm and agricultural income.

	Dependent Variable: Log of per Capita Off-Farm Income			Dependent Variable: Log of per Capita Agricultural Income		
	Model 4	Model 5	Model 6	Model 7	Model 8	Model 9
Training						
Agritech	−0.829	−1.236	−1.107	1.707 ***	0.833 ***	0.695 **
	(−1.018)	(−1.592)	(−1.511)	(6.650)	(3.285)	(2.531)
Offfarmtech	2.398 ***	1.608 **	0.757	−0.158	−0.413	0.010
	(2.847)	(2.387)	(1.227)	(−0.264)	(−0.727)	(0.018)
Human capital						
Age		−0.018	−0.063 **		−0.028 **	0.006
		(−0.730)	(−2.442)		(−2.159)	(0.427)
Gender		1.093	0.294		0.691	0.841 *
		(1.137)	(0.302)		(1.182)	(1.706)
Education		0.702	0.147		0.009	0.195
		(0.995)	(0.235)		(0.023)	(0.521)
Perfeed1		−0.050 ***	−0.048 ***		−0.004	−0.004
		(−5.314)	(−5.579)		(−0.754)	(−0.841)
Social capital						
Party		1.032 *	0.388		0.467	0.427
		(1.776)	(0.712)		(1.432)	(1.362)
Cadre		0.080	0.256		0.158	0.021
		(0.097)	(0.355)		(0.350)	(0.052)
Physical capital						
lnHouseValue		0.728 ***	0.301 **		−0.039	−0.148 *
		(4.556)	(1.987)		(−0.471)	(−1.766)
Road		2.181 **	0.319		−1.190 ***	0.506 *
		(2.333)	(0.285)		(−4.578)	(1.722)
Natural capital						
Forestland		−0.012 *	−0.019 **		0.018 ***	0.009 **
		(−1.652)	(−2.465)		(4.032)	(2.151)
Farmland		0.020 ***	0.035 ***		0.028 ***	0.036 ***
		(2.822)	(4.276)		(5.096)	(5.279)
Biosphere Reserves						
Huangshan			5.288 ***			−2.554 ***
			(6.338)			(−5.484)
Wuyishan			4.078 ***			−1.053 ***
			(4.684)			(−4.073)
Wudalianchi			0.458			−3.423 ***
			(0.459)			(−6.935)
Constant	5.816 ***	3.305 *	6.956 ***	7.046 ***	8.428 ***	7.084 ***
	(19.239)	(1.892)	(3.878)	(38.611)	(11.169)	(8.931)
Observations	381	381	381	381	381	381
R squared	0.017	0.236	0.36	0.037	0.185	0.294

Note: *, **, and *** denote significant mean differences at the 10, 5, and 1 percent levels.

**Table 6:** The impacts of two different types of skill training on household off-farm and agricultural income within and outside PAs.

Variables	Dependent Variable: Log of Per Capita Off-Farm Income		Dependent Variable: Log of Per Capita Agricultural Income	
	Within PA	Outside PA	Within PA	Outside PA
Training				
Agritech	−1.410	−2.113 **	0.770 *	1.029 ***
	(−1.226)	(−2.142)	(1.842)	(2.771)
Offfarmtech	−1.462	0.783*	0.988	−0.127
	(−1.109)	(1.700)	(1.641)	(−0.145)
Human capital				
Age	−0.155 ***	−0.043	0.004	0.016
	(−3.821)	(−1.409)	(0.198)	(0.943)
Gender	−1.423	1.446	1.618 *	0.35
	(−1.021)	(1.197)	(1.872)	(0.598)
Education	0.741	−0.52	0.03	0.454
	(0.508)	(−0.713)	(0.044)	(1.049)
Perfeed1	−0.054 ***	−0.037 ***	−0.010	−0.000
	(−3.981)	(−3.408)	(−1.293)	(−0.059)
Social capital				
Party	0.533	0.678	0.552	0.525
	(0.621)	(1.085)	(1.327)	(1.115)
Cadre	1.023	−0.056	−0.562	0.473
	(0.994)	(−0.062)	(−0.904)	(0.853)
Physical capital				
lnHouseValue	0.047	0.422 *	−0.074	−0.273 *
	(0.255)	(1.907)	(−0.654)	(−1.935)
Road	−7.545 ***	4.721 ***	2.332 ***	−0.367
	(−4.039)	(3.175)	(3.709)	(−0.774)
Natural capital				
Forestland	0.005	−0.004	−0.003	0.009
	(0.338)	(−0.632)	(−0.408)	(1.448)
Farmland	0.018	0.037 ***	0.039 ***	0.033 ***
	(1.412)	(3.581)	(3.403)	(3.398)
Biosphere Reserves				
Huangshan	10.234 ***	3.324 ***	−2.062 **	−2.762 ***
	(5.929)	(3.898)	(−2.470)	(−5.215)
Wuyishan	9.000 ***	1.807 *	−1.226 ***	−1.224 ***
	(6.292)	(1.832)	(−2.683)	(−3.058)
Wudalianchi	6.163 ***	−0.575	−4.042 ***	−3.659 ***
	(3.497)	(−0.448)	(−4.854)	(−4.658)
Constant	16.733 ***	1.677	5.483 ***	7.911 ***
	(6.128)	(0.793)	(3.561)	(8.858)
Observations	158	223	158	223
R squared	0.467	0.392	0.396	0.298

Note: *, **, and *** denote significant mean differences at the 10, 5, and 1 percent levels.

## Data Availability

Not applicable.
